# Total Hip Replacement with a Fully Hydroxyapatite-Coated Shortened Stem: Five- to Thirteen-Year Follow-Up Results

**DOI:** 10.3390/jcm13092657

**Published:** 2024-05-01

**Authors:** Fernando Marqués López, Ivet Pares Alfonso, Daniel Donaire Hoyas, Gregorio Ruiz Morales, Marc Tey Pons, Xavier Lizano Díez, Alfonso León García

**Affiliations:** 1 Parc de Salut Mar, 08003 Barcelona, Spain; ipares@psmar.cat (I.P.A.); gmorales@psmar.cat (G.R.M.); mtey@psmar.cat (M.T.P.); xlizano@psmar.cat (X.L.D.); aleon@psmar.cat (A.L.G.); 2 Hospital Universitario Poniente, 04700 Almería, Spain; ddhmp5@hotmail.com

**Keywords:** hip, arthroplasty, replacement, orthopedics, short stem, total hip arthroplasty

## Abstract

**Background**: Shortened femoral stems aim to mimic the biomechanical performance of traditional stems while preserving more bone and minimizing soft tissue damage. Our objective is to assess the outcomes of patients treated with a shortened stem (Furlong Evolution, JRI Orthopaedics, Sheffield, UK) to analyze the implant’s efficacy and survivorship. **Methods**: This retrospective observational study included all patients aged 18 to 70 undergoing uncemented shortened stem total hip replacement at Hospital del Mar between 2010 and 2018. Hip function and pain were assessed with the Merle d’Aubigné–Postel scale and visual analog scale, respectively. A radiographic analysis measured stem and cup orientation, leg length discrepancy, stem subsidence, and radiolucencies around the cup. Perioperative complications, prosthetic failures, and reoperations were documented. **Results**: A total of 109 patients (74 male, 35 female) of a mean age of 51.8 ± 8.8 years were included. The average follow-up was 91 ± 17.4 months. Radiographically, 71 (65.1%) of the stems had been inserted at the appropriate angulation (±3°), and 102 (93.6%) of the cups had been placed in the Lewinnek safety zone. Leg length discrepancy was observed in 19 (17.4%) cases. The mean Merle d’Aubigné–Postel score improved from 13.1 ± 1.39 preoperatively to 17.8 ± 0.49 at 6 months postoperatively (*p* < 0.001). Merle d’Aubigné–Postel subscales also reflected a statistically significant improvement (*p* < 0.001). The mean pain score 12 months postoperatively was 0.52 ± 1.22, with 95.4% of patients declaring themselves satisfied or highly satisfied. The expected 13-year survival according to a Kaplan–Meier analysis was 100% in the absence of infection and 91.3% if revision for any cause is taken as a survival endpoint. **Conclusions**: The shortened stem under analysis provides excellent functional results and long-term survival rates.

## 1. Introduction

Total hip replacement (THR) has been hailed as the surgical procedure of the 20th century [1]. Indeed, it has over time become a highly effective standardized procedure capable of eradicating pain and restoring function in patients with degenerative hip conditions. In addition, the survival of the prosthetic components has improved in sync with technological advancements [2]. However, the type of patients undergoing THR has also evolved [3,4,5], as have their expectations [1].

THR has gone from being a last-resort procedure reserved for elderly patients to becoming a valid option for active young individuals wishing to preserve their quality of life despite their disease [3,4,5]. Consequently, although the results of THR are outstanding [2], the higher levels of activity of patients currently undergoing the procedure mean that prosthetic components are subject to higher levels of mechanical stress, often at the same time as other well-known problems, such as periprosthetic fractures [6,7], thigh pain [7,8,9,10], proximal stress shielding [7,10,11,12,13], or bone loss [7,10].

The response to these challenges has been the development of the so-called short stems, a heterogeneous category that encompasses a wide variety of prosthetic designs [13,14,15,16], all of which, however, share common goals, including preserving the patient’s bone stock, facilitating subsequent revisions with a conventional stem, and, theoretically, providing for an ideal fit with the typical femoral geometry of young individuals [13,14,17,18,19,20] (Dorr type A femora [21]). Clinical experience with short stems is, however, still limited. In addition, neck-preserving designs require a more complex surgical technique [16,18,22,23] and are less tolerant of errors in size selection, placement, or the level of bone resection [14]. Indeed, some such models have been associated with high rates of malalignment [11]. These drawbacks have led several surgeons to recommend prudence in the use of short stems or that they be reserved for young patients with good bone quality [14,16,24,25]. Against this background, the so-called shorter stems burst onto the scene as a way of capitalizing on the advantages of short stems and surmounting their weaknesses. These designs, which are a shorter version of traditional stems [16], were conceived with the aim of preserving bone stock and allowing for a less aggressive surgical approach while replicating the biomechanical behavior of traditional stems [26]. A clinical comparison between a conventional stem and its shortened version would allow us to analyze if the different geometry involves different results.

The Furlong Evolution stem is a shorter version [16] of the Furlong H-A.C. stem (JRI Orthopaedics, Sheffield, UK) [26,27], a fully hydroxyapatite-coated conventional stem that has demonstrated high long-term survival rates [28,29]. In order to find out if the advantages attributed to shorter stems are actually reflected in clinical practice, our objective is to assess the outcomes of patients treated with such a shortened stem to analyze its medium-term (5- to 13-year) survival and efficacy. Our hypothesis is that the clinical outcomes should be similar to those of the conventional stem.

## 2. Materials and Methods

### 2.1. Overview

The study was approved on the 25th of February 2015 by the Centro Almería Ethics Committee, and all patients gave their informed consent before they were included. The analysis included all patients undergoing a THR with the Furlong Evolution short stem (JRI Orthopaedics, Sheffield, UK) in our hospital between 1 October 2010 and 31 October 2018. Patients under 18 and over 70 years of age were excluded from the study, as were those diagnosed with cancer, those on chronic treatment with corticosteroids or immunosuppressants, those with senile dementia, and those with alcohol and/or drug abuse problems. Patients presenting with Dorr type C femoral morphologies [21] were also excluded in the study. All subjects gave their informed consent to participate in the study.

### 2.2. Implants

**Figure 1:**
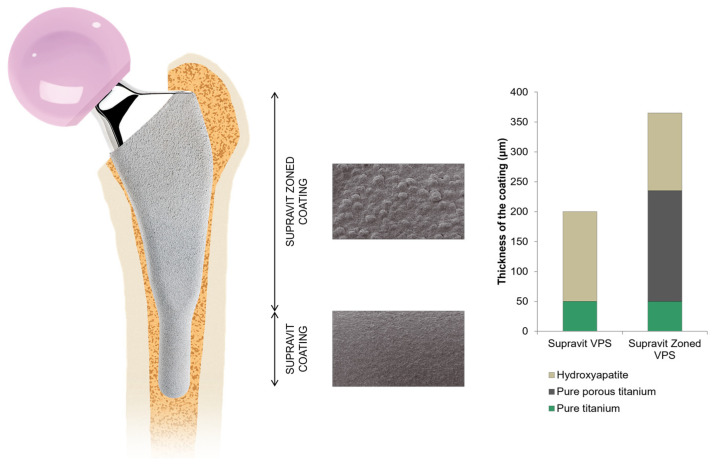
Furlong Evolution stem (JRI Orthopaedics, Sheffield, UK).

The Furlong Evolution stem (Figure 1) is a shorter version of the Furlong H-A.C. stem (JRI Orthopaedics, Sheffield, UK), the first fully hydroxyapatite-coated stem ever developed [27], which has demonstrated survival rates of 91.7% at +20 years (100% if infection is excluded as a cause of failure) [28,29].

**Figure 2:**
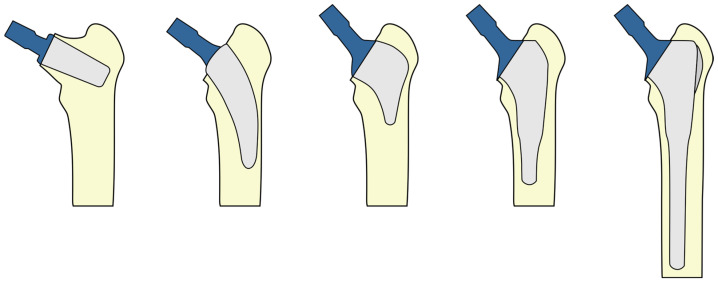
Types of short stems according to Khanuja’s classification [16]. From left to right: (I) stem with femoral neck fixation; (II) calcar loading stem; (III) calcar loading stem with lateral flare; (IV) shortened conventional stem; (V) conventional stem.

**Figure 3:**
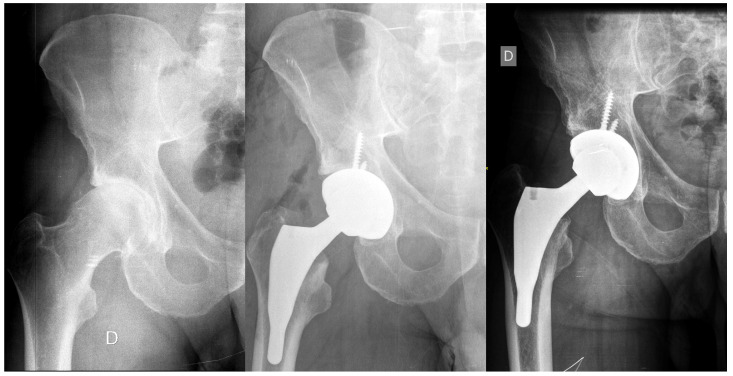
AP X-rays displaying the (I) preoperative condition, (II) immediate postoperative status, and (III) 5-year follow-up of a patient treated with an Evolution hip stem.

The Furlong Evolution stem, which is a type IV stem (shortened conventional stem) according to Khanuja’s classification [16], has been shown to exhibit a biomechanical performance in a finite element analysis similar to that of its conventional predecessor [26] (Figure 2 and Figure 3), and it is commercially available.

### 2.3. Surgical Technique

All the procedures analyzed in this study were conducted by three expert hip surgeons, with an accumulated experience of over 15 years with the conventional Furlong H-A.C. stem. A modified Hardinge approach was employed in all cases, with the patient in the lateral position. As required by the hospital’s protocol, patients received preoperative prophylaxis, intraoperative tranexamic acid (if not contraindicated), and postoperative antithrombotic therapy. As regards rehabilitation, the hospital’s THR protocol was followed, which included assisted walking with two crutches from the second day post-op as well as abductor muscle strengthening exercises. Unassisted walking was started at one month from surgery.

### 2.4. Variables

A record was made of the patients’ anthropometric data (age, sex, body mass index [BMI], and ASA score), their underlying diagnosis, and the duration of follow-up. Function was evaluated by means of the Merle d’Aubigné–Postel (MDP) score [30,31,32], which was calculated preoperatively and at 6 months from surgery, as it has already been shown that, from that moment on, the improvements that occur are not relevant [33,34,35,36]. Pain was assessed through the visual analog scale (VAS) at 12 months from surgery. The MDP score evaluates pain, mobility, and gait in three subscales ranging from 0 to 6 and on a global scale that adds them, while the VAS evaluates pain ranging from 0 (painless) to 10 (maximum pain imaginable). A radiological study was carried out of the orientation of both the stem and the cup, checking for signs of leg-length discrepancy, stem subsidence, and radiolucency around the cup. Intraoperative complications as well as those occurring during follow-up were recorded, as were any prosthetic failures and reoperations.

### 2.5. Statistical Analysis

A descriptive analysis of the data was carried out using measures of central tendency and dispersion. The comparison between preoperative and postoperative values was performed using repeated measures of variance. Differences between the groups were analyzed using a one-way ANOVA or the Kruskal–Wallis test, depending on the fulfillment of the required statistical assumptions. Qualitative variables were analyzed using Pearson’s chi-square test or the Fisher exact test, depending on the magnitude of the values expected. Survival was assessed following the Kaplan–Meier method, including all the stems implanted between the aforementioned dates even if the minimum follow-up period had not been reached. In all cases, a *p*-value of 0.05 was considered statistically significant. The analysis of data was conducted using R software v. 4.1.3. (R Development Core Team) [37].

## 3. Results

A total of 109 patients (74 males and 35 females) were included in the study, with a mean follow-up of 91 ± 17.4 months (range: 60–151). The mean patient age was 51.8 ± 8.8 years, mean height was 169 ± 8.8 cm, and mean BMI was 27.3 ± 4.5. The subjects’ anthropometric and surgical data are summarized in Table 1.

The most common underlying diagnosis was osteoarthritis (N = 78; 71.6%), followed by avascular necrosis (N = 17; 15.6%), dysplasia (N = 7; 6.4%), rheumatoid arthritis (N = 2; 1.8%), and fractures (N = 1; 0.9%). The four remaining cases (3.7%) exhibited multiple other underlying diagnoses. Most patients (3.7%) were classified as ASA I (N = 52; 47.7%) and ASA II (N = 48; 44.0%), with only eight cases of ASA III (7.3%) being identified.

The mean surgical time was 58.8 ± 12.5 min (range: 38–96), with a mean blood loss of 298.0 ± 99.5 mL. Three patients required a blood transfusion after the procedure.

**Figure 4:**
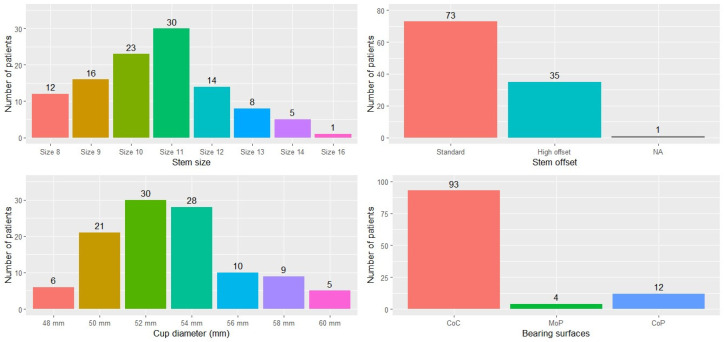
Distribution of the different implant sizes and bearing surfaces used.

The implants used in the different procedures are shown in Figure 4. The most common stem size was size 11, with 73 (67.0%) stems featuring a standard offset design and 35 (32.1%) an extended offset design. As regards the cup, the most commonly used diameters were 52 mm (N = 30; 27.5%), 54 mm (N = 28; 25.7%), and 50 mm (N = 21; 19.3%), followed at great distance by the remaining sizes. The most commonly used bearing surface was ceramic-on-ceramic (CoC; N = 93; 85.3%), followed by ceramic-on-polyethylene (CoP; N = 12; 11.0%) and metal-on-polyethylene (MoP; N = 4; 3.7%).

Three patients developed complications during the surgical procedure (two acetabular fractures and one calcar crack), which did not require reoperation. As regards early complications, there were five hematomas, three cases of heart failure, one acute febrile syndrome, and one superficial femoral artery pseudoaneurysm.

X-ray analyses revealed that 71 (65.1%) stems had been implanted at the right angulation, whereas 35 (32.1%) had been implanted in varus and three (2.8%) in valgus. As far as the cups are concerned, 102 (93.6%) were implanted in the safety zone, whereas seven (6.4%) showed an excessively vertical placement. Signs of leg-length discrepancy were observed in 19 (17.4%) cases. The mean discrepancy in these cases was 6.3 ± 4.9 mm (range: 1–15), rendering them clinically irrelevant. Seven (6.4%) cups exhibited radiolucencies, two of them in zone I, two in zone II, and three in all Gruen zones.

**Figure 5:**
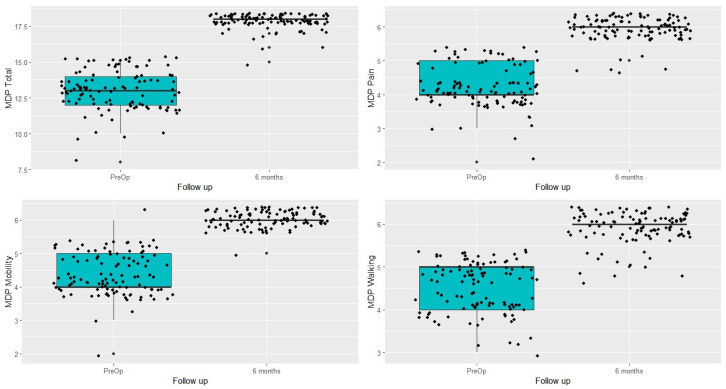
Evolution of the different Merle d’Aubigné–Postel subscales between the preoperative period and sixth months post-op.

The evolution of the patients’ clinical status between the preoperative period and the sixth month post-op, as measured by the MDP scale, is summarized in Table 2 and Figure 5. Both the overall assessment and the three MDP subscales (pain, gait, and mobility) showed a considerable (and statistically significant) clinical improvement (*p* < 0.001).

Follow-up data are summarized in Table 3. The mean pain score at 12 months post-op, as measured by the VAS scale, was 0.52 ± 1.22. Twelve patients (11.0%) developed Greater Trochanter Pain Syndrome, but no cases of thigh pain were identified.

A total of five (4.6%) complications arose during follow-up, including two greater trochanter fractures, one dislocation, one nerve lesion, and a hematoma. All of them were resolved without the need of reoperation. In addition, squeaking was documented in seven cases (6%).

**Figure 6:**
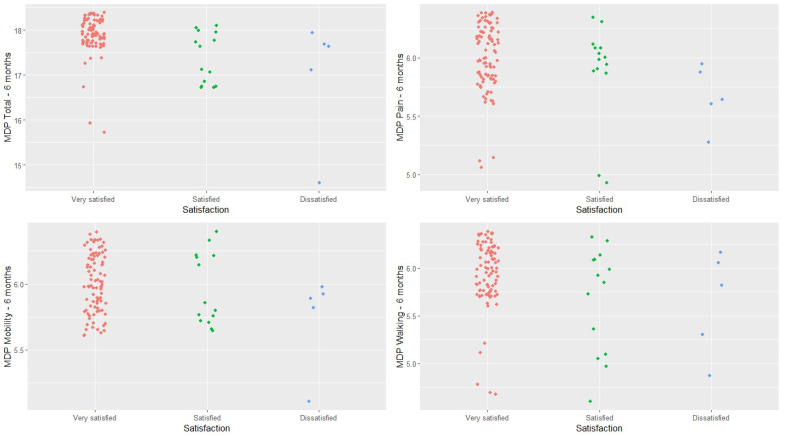
Relationship between patient satisfaction and patient function at 6 months as measured by the Merle d’Aubigné–Postel scale.

A total of 95.4% of patients declared themselves to be satisfied or highly satisfied with the result of the procedure. The remaining 4.6% declared themselves dissatisfied, with no patient declaring themselves highly dissatisfied. Patient satisfaction was correlated (Figure 6) with the pain score at 6 months (*p* < 0.001), where certain differences were found between the “highly satisfied” group and the other two groups (*p* < 0.001 in both cases).

A connection was also found between patient satisfaction and some of the scores on the MDP scale at 6 months (not considering the baseline scores). More specifically, statistically significant differences were found between the different levels of satisfaction reported by patients and their overall MDP score. A statistically significant difference was observed (*p* < 0.001) between “highly satisfied” and “satisfied” patients as well as between “highly satisfied” and “dissatisfied” patients (*p* = 0.016). No differences were observed (*p* = 0.087) between the different levels of satisfaction and the scores obtained on the MDP pain subscale. As regards the mobility subscale, differences were observed (*p* < 0.001) between the “highly satisfied” and the “dissatisfied” group. Significant differences were also found in terms of the MDP gait subscale. Such differences were observed between the “highly satisfied” and “satisfied” groups (*p* = 0.001) and the “highly satisfied” and “dissatisfied” groups (*p* = 0.009).

**Figure 7:**
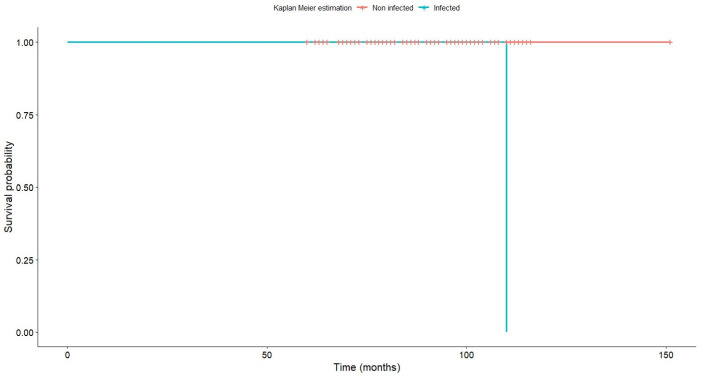
Implant survival according to the Kaplan–Meier methodology. A distinction is made between infected and non-infected prostheses.

As far as implant survival is concerned, none of the stems had to be revised for aseptic loosening. Two stems, however, had to be revised for infection. This means that the expected 13-year survival according to the Kaplan–Meier methodology was 100% in the absence of infection, and 91.3% if revision for any cause is taken as a survival endpoint (Figure 7).

## 4. Discussion

The most significant finding of our analysis was that the Furlong Evolution shortened stem showed excellent functional results and a very high survival rate, in line with that of traditional stems [2]. None of the components in our series had to be revised for aseptic loosening, and only two failed for septic reasons. In addition, the functional improvement experienced by the patient was clinically and statistically significant, with very high satisfaction scores. These results are particularly notable given that our cohort comprised relatively young patients (mean age: 51.8 ± 8.8 years), whose high levels of activity constitute a significant challenge for the survival of these implants. Moreover, the mean follow-up was 7.6 years, which is longer than in any study on the stem under analysis.

Both the registry data [2] and the medical literature agree that the survival of the longer version of the studied stem is currently very long. Sandifords et al. [28] reported survival rates for aseptic loosening of 100% (91.7% in the case of survival for any cause) in 72 Furlong H-A.C. stems with a mean follow-up of 22.5 years. Using the same implant, Rajaratnam et al. [29] also obtained a 100% survival rate for aseptic loosening (97.4% considering all potential causes of revision) in 331 stems with a mean follow-up of 17.5 years. Identical aseptic survival rates were reported by Pertegàs et al. [38] in a series of 254 stems with 10 or more years’ follow-up. Other authors obtained similar results even in younger patient series. In their series of 38 Furlong H-A.C. stems implanted in patients under 50 years of age, Syed et al. [22] obtained aseptic survival rates of 100% with follow-up periods between 17 and 25 years. In the same vein, Robertson et al. [39] presented a 14-year survival rate of 95.3% in 68 conventional stems implanted in patients under 55 years of age.

Considering the encouraging survival rates of the Furlong H-A.C. stem, one might be justified in supporting the addition of short stems to the orthopedic surgeon’s therapeutic arsenal. However, what do we mean by a short stem? Although all the designs that fall under that category seek to conform to the characteristics and expectations of the new patients presented to our orthopedic departments [1,3,4,5], it must be considered that not all of them share the same biomechanical profile [16]. In this connection, the stem analyzed in this study is a Khanuja type IV stem (shortened conventional stem) [16], which has been shown to exhibit a similar biomechanical performance to that of its predecessor [26] and should therefore present with similar survival rates. Moreover, it has been demonstrated that the stem’s hybrid design allows for bone stock preservation [40] and does not affect the patients’ gait [41] or result in a higher risk of migration or subsidence [42]. In fact, the subsidence rates of the Furlong Evolution stem on the UK National Joint Registry are practically identical to those of its predecessor [2], with a 5-year revision rate of 2.01%, as compared with 2.0% for the traditional Furlong H-A.C. stem.

This study found a 13-year survival rate of 100% if aseptic failure is taken as an endpoint. As two patients in our series had to be reoperated due to an infection of the implant, the survival rate (defining failure as revision for any cause) was 91.3%. These figures, which are in line with those reported for the traditional Furlong stem, are highly encouraging, not least considering that patients in our study were relatively young and therefore subjected their stems to higher biomechanical stresses.

The functional results obtained for the Furlong Evolution stem were also very promising, with 106 (97.2%) patients achieving very good functional results and another three achieving good functional results according to the grading proposed by Sahin et al. [32] based on the MDP scale. These results are superior to those reported by Sandiford et al. [28], although it must be acknowledged that these authors conducted the evaluation at the end of the follow-up period rather than at six months, as in our case, which means that patient aging could have negatively impacted their results. Robertson et al. [39] reported a median MDP score of 17, which is also lower than the MPD score in the present series, which stood at 18 points. Other analyzed studies used different functional grading scales, which makes direct comparisons more difficult. It should be highlighted that the shortened stem appears to be highly forgiving of technical errors, as no clinically relevant or statistically significant differences (*p* = 0.072) were found when comparing the subgroup of patients with well-aligned stems with patients exhibiting malalignment. Specifically, the percentage of malaligned stems in our study was 34.9%, which is not too far from the 30% reported by Pertegàs et al. [38] in their analysis of conventional stems. Like us, these authors did not find any functional differences between patients with well- and poorly-aligned stems. As the Furlong Evolution stem is a shortened version of the Furlong H-A.C. stem, the surgical technique used to insert it is similar to that employed with its predecessor. However, the morphology of the shortened stem allows for a better fit into the medullary canal, reducing the incidence of malpositioning as compared with other short stems accused of being unforgiving of technical errors and malalignment [11,14].

As far as patient satisfaction is concerned, our results are comparable to those reported by studies on traditional stems. A total of 92% of patients in Sandiford et al. [28] declared themselves highly satisfied, and 71.7% of those in Pertegàs et al. [38] reported excellent satisfaction levels. Our results, with 82.6% of highly satisfied patients, are in-between those obtained by Sandiford et al. and Pertegàs et al.

The mean surgical time in our series was 58.8 min, and the most remarkable intraoperative complications were two acetabular fractures and a crack in the calcar, all of which were resolved without the need of reoperation. This would appear to contradict the conclusions of some studies that accuse short stems of demanding a more complex surgical technique [16,18,22,23]. Nevertheless, one should not lose light of the heterogeneity of prosthetic designs discussed in the different studies. In our analysis, two fractures of the greater trochanter and one dislocation occurred during follow-up, both of them resolving without the need of surgery. Trochanteritis was observed in 12 (11.0%) patients, but no cases of anterior thigh pain were identified, without any correlation being found between the presence of trochanteritis and the VAS pain score (*p* = 0.263). Our rate of complications was lower than that obtained by Pertegàs et al. [38], who reported nine intraoperative greater trochanter cases and 16 calcar fractures in addition to eight dislocations, although it must be said that the complications faced by these authors were most probably attributable to the steep learning curve associated with the Furlong Evolution stem, which we had already navigated to a certain extent thanks to our experience with the traditional stem.

Our study is not exempt from limitations, the main one being the lack of randomization and the fact that no comparison is made with other treatment alternatives. We would also like to advise researchers that the clinical evaluation was performed at six months instead of the usual one-year mark. Nonetheless, this deviation has been duly justified in Section 2. Nonetheless, we believe that as our goal was to determine the efficacy and safety profile of the analyzed short stem design, the observational character of the study together with the abundant comparisons made with the results reported in the literature constitute validation enough to answer our research question. Moreover, this study constitutes the largest and longest followed-up series to date on the Furlong Evolution stem. The follow-up period of the study is indeed long enough to warrant the extraction of valid conclusions regarding both function and survival. In fact, our experience confirms that shortened stems are a safe and effective option. However, we want to be cautious and we must recognize that new studies will be necessary before we can generalize this option to all types of patients, although some authors have already shown good results even in obese patients [43,44].

## 5. Conclusions

The shortened Furlong Evolution stem is associated with a substantial improvement in functional outcomes and a low incidence of complications in addition to long-term survival rates comparable to those of the conventional Furlong H-A.C. stem. As a result, this stem can be considered a valid alternative for active young patients, who are likely to subject the prosthesis to severe functional demands and who may undergo one or more prosthetic revisions in their lifetime.

## Figures and Tables

**Table 1:** Anthropometric and surgical data.

Variable	N	%	Mean	SD	Min	Max
Sample size	109	-	-	-	-	-
Anthropometrics						
Sex (M/F)	74/35	68%/32%	-	-	-	-
Age (years)	109	-	51.8	8.82	25	67
Weight (kg)	109	-	78.5	14.5	53	120
Height (cm)	109	-	169	8.1	150	187
BMI	109	-	27.3	4.46	20.2	40.8
Underlying diagnosis						
Osteoarthritis	85	78.0%	-	-	-	-
Avascular necrosis	17	15.6%	-	-	-	-
Other	4	3.7%	-	-	-	-
Rheumatoid arthritis	2	1.8%	-	-	-	-
Fracture	1	0.9%	-	-	-	-
ASA score						
ASA I	48	44.0%	-	-	-	-
ASA II	52	47.7%	-	-	-	-
ASA III	8	7.3%	-	-	-	-
Surgery						
Surgical time	109	-	58.8	12.5	38	96
Blood loss (ml)	109	-	298.0	99.5	100	600
PRBCs (units)						
0 units	106	97.2%	-	-	-	-
1 unit	2	1.8%	-	-	-	-
2 units	1	0.9%	-	-	-	-
Follow-up (months)	109	-	91.0	17.4	60	151

**Table 2:** Merlé D’Aubigné–Postel score.

	PreOp	6 Months	*p*-Value
Pain	4.19 ± 0.57	5.94 ± 0.23	<0.001 *
Mobility	4.38 ± 0.59	5.99 ± 0.10	<0.001 *
Gait	4.49 ± 0.59	5.89 ± 0.31	<0.001 *
Total score	13.1 ± 1.39	17.8 ± 0.49	<0.001 *

* Statistically significant differences are detected between pre- and post-surgery values.

**Table 3:** Follow-up data.

Variable	N	%	Mean	SD	Min	Max
Pain—6 month-VAS	109	-	0.52	1.22	0	6
Squeaking	7	6.4%	-	-	-	-
Complications						
None	104	95.4%	-	-	-	-
Dislocation	1	0.9%	-	-	-	-
Nerve lesion	1	0.9%	-	-	-	-
Hematoma	1	0.9%	-	-	-	-
Greater trochanter fracture	2	1.8%	-	-	-	-
Other problems						
Trochanteric pain	12	11.0%	-	-	-	-
Cup loosening	3	2.8%	-	-	-	-
Stem subsidence	2	1.8%	-	-	-	-
Cup radiolucencies						
None	102	93.6%	-	-	-	-
Zone I	2	1.8%	-	-	-	-
Zone II	0	0.0%	-	-	-	-
Zone III	2	1.8%	-	-	-	-
All zones	3	2.8%	-	-	-	-
Satisfaction						
Highly satisfied	90	82.6%	-	-	-	-
Satisfied	14	12.8%	-	-	-	-
Dissatisfied	5	4.6%	-	-	-	-
Highly dissatisfied	0	0.0%	-	-	-	-

## Data Availability

Dataset available on request from the authors.
